# Reinforcement Effects on Tensile Behavior of Ultra-High-Performance Concrete (UHPC) with Low Steel Fiber Volume Fractions

**DOI:** 10.3390/ma17102418

**Published:** 2024-05-17

**Authors:** Xianzhi Luo, Sumei Zhang, Aidong Li, Chenming Zhang, Yuchen Zhang

**Affiliations:** 1 School of Civil and Environmental Engineering, Harbin Institute of Technology, Shenzhen, University Town, Shenzhen 518055, China; luoxianzhi_hit@163.com (X.L.); yczhang_hit@163.com (Y.Z.); 2 Guangdong Provincial Key Laboratory of Intelligent and Resilient Structures for Civil Engineering, Shenzhen 518055, China; 3 China Railway Design Corporation, Tianjin 300308, China; liaidong@crdc.com (A.L.); zhangchenming@crdc.com (C.Z.)

**Keywords:** ultra-high-performance concrete (UHPC), fiber volume fraction, rebar, ductility, cracking behavior, direct tensile test, digital image correlation (DIC)

## Abstract

Ultra-high-performance concrete (UHPC) with a low steel fiber volume fraction offers lower material costs than UHPC with typical steel fiber volume fractions, and has the potential to mitigate the ductility degradation of rebar-reinforced UHPC (R-UHPC). This study explores the reinforcement effect on the tensile behavior of UHPC with a low fiber volume fraction with the aim of facilitating more cost-efficient UHPC applications. The axial tensile behavior of 30 UHPC specimens with low fiber volume fractions at different reinforcement ratios was tested through direct tensile tests. The findings indicate that adopting UHPC with a low fiber volume fraction can significantly mitigate the ductility deterioration of rebar-reinforced UHPC (R-UHPC), and both increasing the reinforcement ratio and decreasing the fiber volume fraction contribute to the improvement in ductility. The failure modes of R-UHPC are determined by the ratio of reinforcement ratio and fiber volume fraction, rather than a single parameter, which also means that R-UHPC with different parameters may correspond to different methods to predict tensile load-bearing capacity. For UHPC with a fiber volume fraction low to 0.5%, incorporating steel rebars gives superior multi-crack cracking behavior and excellent capacity to restrict the maximum crack width. Increasing the fiber volume fraction from 0.5% to 1.0% at the same reinforcement ratio will yield little benefit other than an increase in tensile load-bearing capacity.

## 1. Introduction

**Figure 1:**
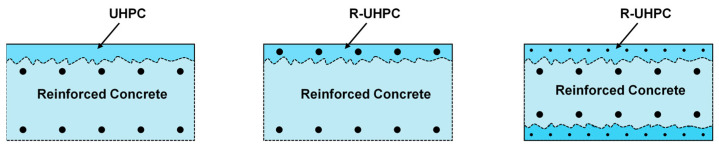
UHPC is adopted as a protective or strengthening layer.

Ultra-high-performance concrete (UHPC) is a superior cement-based material, especially in terms of tensile behavior and durability [1,2]. Owing to these attributes, UHPC has emerged as the material of choice for augmenting the mechanical properties and durability of engineering structures subjected to severe service environments [3,4]. It finds application in bridge decks for withstanding substantial and complex loads [5,6], in underground structures to impede water intrusion [7,8], and in strengthening existing structures to bolster their load-bearing capacity and extend their service life [9,10]. However, the considerable use of steel fibers substantially escalates the cost of UHPC. The material cost of UHPC with a 2% fiber volume fraction is roughly 10 to 20 times higher than that of ordinary concrete, with steel fibers representing 30% to 50% of the total expenditure. This cost factor limits its application to certain critical components [11,12], such as reinforcement and protective layers for structural members, as shown in Figure 1.

Reducing the steel fiber volume fraction leads to a substantial reduction in the material cost of UHPC. However, it often triggers an overall decline in tensile behavior, including tensile strength, toughness, and multi-cracking behavior. A fiber volume fraction of less than 2% can be defined as a low fiber volume fraction [3,13,14]. Through direct tensile testing of UHPC, Liu et al. [15] demonstrated that a reduction in fiber volume fraction from 2.5% to 1.75% and 1.0% led to a corresponding decrease in tensile strength from 8.10 MPa to 6.41 MPa and 5.85 MPa, respectively. Similarly, Ren et al. [16] explored the tensile behavior of UHPC with different fiber volume fractions, discovering a reduction in fracture energy by 34.1% and 71.4% when the fiber volume fraction decreased from 2.5% to 1.5% and 0.5%, respectively, with a significant deterioration in tensile ductility. Investigations on the response of UHPC under axial tensile load by Qiu et al. [17] and Yang et al. [18] found that UHPC with lower fiber volume fraction demonstrated a rapid decrease in load-bearing capacity after cracking, and virtually no secondary cracks developing on either side of the main fracture surface. The detrimental effect of reducing fiber volume fraction on the tensile performance of UHPC has been further supported by additional studies [19,20,21,22,23].

Integrating steel rebar into UHPC has been demonstrated to enhance its tensile response, including mechanical properties and cracking behavior, even at low fiber volume fractions. Research conducted by Aghdasi et al. [24] and Kunieda et al. [25] comparing the axial tensile behavior of UHPC before and after the addition of rebar, found that even a modest rebar inclusion significantly enhances the ductility of UHPC, and the ductility of UHPC post rebar inclusion reaches nearly 7.5 times that of pre-rebar inclusion. Bian et al. [26] and Qiu et al. [27] tested the direct tensile behavior of UHPC and R-UHPC and suggested that the inclusion of rebars enhances the post-cracking stiffness and load-bearing capacity of UHPC, and simultaneously restrains the maximum crack width propagation. Guo et al. [28] and Luo et al. [29] further studied the tensile ductility of UHPC and R-UHPC, revealing that UHPC with a lower fiber volume fraction exhibits superior ductility at the same reinforcement ratio. Indeed, to meet the structural load-bearing requirements, the incorporation of steel rebars within UHPC has become common practice in engineering.

However, some studies have indicated that members constructed of R-UHPC tend to display diminished tensile and flexural ductility compared to their counterparts constructed of ordinary reinforced concrete, and are inclined towards brittle failure. Both Hung et al. [30] and Kang et al. [31] performed deformation performance tests on R-UHPC with varying reinforcement ratios, asserting that the tensile ductility of R-UHPC is substantially inferior to that of bare rebar. Moreover, R-UHPC with a smaller reinforcement ratio has a higher propensity to succumb to premature tension failure. Experimental demonstrations by Shao et al. [32,33], Yang et al. [34], and Hasgul et al. [35] conveyed that R-UHPC flexural members with common reinforcement ratios often fail due to rebar fracture subsequent to crack localization and that employing lower fiber volume fractions and higher reinforcement ratios can potentially mitigate the issues associated with brittle damage. Similarly, tests on R-UHPC beams by Turker et al. [36] and Yoo et al. [37,38] indicated that while the addition of steel fibers enhances the load-bearing capacity of the beam and provides superior cracking behavior, it simultaneously induces a significant deterioration in ductility and the addition of 2% fibers leading to a maximum of a 73% reduction in ductility.

Despite the advantages of lower material costs and the potential to enhance the ductility of R-UHPC members attributed to UHPC with a low fiber volume fraction, existing research predominantly concentrates on UHPC with typical fiber volume fractions ranging from 2.0% to 3.0%. Studies focusing on UHPC with a low fiber volume fraction are scarce, and when such topics are mentioned, they often lack systematic analysis as they are not the main subjects of investigation. For instance, the effectiveness of improving the tensile ductility of R-UHPC members using UHPC with a low fiber volume fraction has only been discussed in a relatively cursory manner, and a comprehensive evaluation of the impact of changing the reinforcement ratio on tensile ductility has not yet been performed. Furthermore, when employing R-UHPC as a protective layer for structures, its tensile cracking behavior directly influences the durability of the structure. However, current research concerning the tensile cracking behavior of R-UHPC with a low fiber volume fraction remains inadequate. Therefore, to better guide the selection of material parameters and facilitate the rational, cost-efficient, and broader application of UHPC, having an insight into the impact of varying the reinforcement ratio on its mechanical properties and cracking behavior holds great importance.

This paper focuses on UHPC with low fiber volume fractions, investigating the impact of varying the reinforcement ratio on its tensile mechanical properties and cracking behavior. The tested reinforcement ratios encompass 0%, 1.7%, 3.0%, 4.7%, and 6.8%, and the fiber volume fractions are 0.5% and 1.0%. The appraisal of mechanical properties primarily delves into the failure mode, load-displacement relationship, and mechanical feature points of UHPC, and the analysis of cracking behavior predominantly focuses on the cracking process and crack characteristics. Concurrently, the efficiency of fibers in UHPC with different parameters in enhancing mechanical properties and improving cracking behavior is assessed. In addition, the effects of the rebar layout, which changes with the reinforcement ratio, on fiber distribution characteristics are explored.

## 2. Materials and Methods

### 2.1. Material Property

The UHPC utilized for testing was a commercial product provided by specialized suppliers in Ningbo, China, encompassing premixed powder, superplasticizer, and steel fibers. Owing to the superior equilibrium of mechanical properties and workability offered by mixed fibers [39,40], the fibers utilized in the experiment consisted of a 1:1 combination of straight and hooked-end fibers. The diameters of the straight and hooked-end fibers measure 0.20 mm and 0.22 mm, respectively, and both types of fibers have a length of 16 mm and a nominal strength of 2800 MPa. The fiber volume fractions were set at two levels, 0.5% and 1.0%. The curing and mechanical property testing of UHPC were conducted following the guidelines outlined in the Chinese specification GB/T 31387. Remarkably, despite a 50% increase in fiber volume fraction from 0.5% to 1.0%, there is no corresponding enhancement in UHPC tensile strength, compressive strength, and elastic modulus, as summarized in Table 1. This is primarily attributable to the fact that a fiber volume fraction of 1.0% still falls short of providing a sufficient bridging effect on the crack surface to trigger strain hardening.

**Figure 2:**
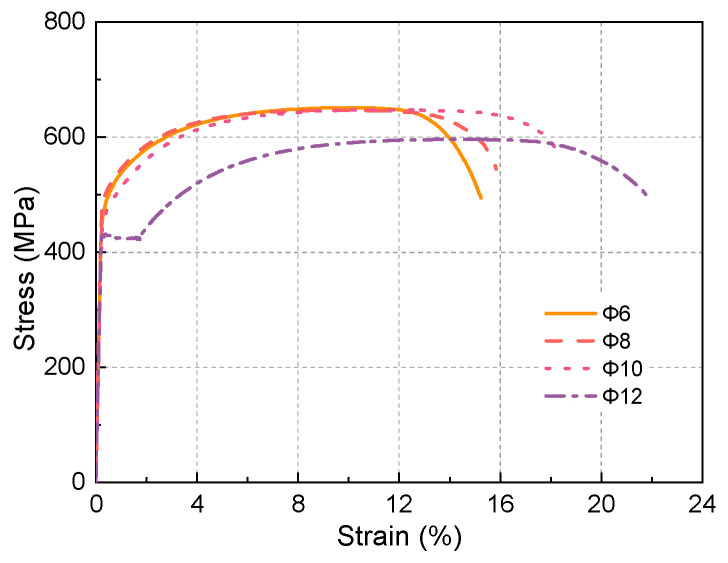
Stress–strain curves of HRB400 steel rebar with different diameters.

Four different diameters of HRB400 bars (6 mm, 8 mm, 10 mm, and 12 mm) were employed in the experiments. The mechanical properties of the rebar were tested according to the Chinese specification GB/T228.1 [41] and the stress–strain curves are shown in Figure 2 and the essential performance parameters are cataloged in Table 2. It is noteworthy that the rebar with a 12 mm diameter, due to its particular yield plateau, exhibited superior fracture ductility compared to the other diameters, as illustrated in Figure 2. This disparity in performance among different diameter rebars aligns with engineering practice. Typically, rebars with diameters smaller than 12 mm are transported as bar coils and require straightening prior to usage, a process that induces plastic deformation and consequent loss of the yield plateau.

### 2.2. Specimen Design

**Figure 3:**
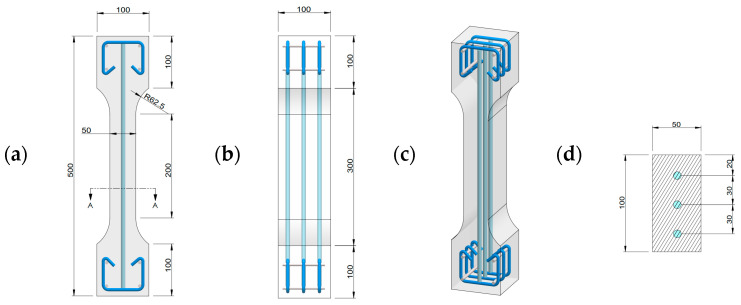
R-UHPC specimen details: (**a**) front view; (**b**) side view; (**c**) isometric view; (**d**) A-A section view [42].

The specimen specifications used in this study are illustrated in Figure 3. The specimen was a rectangular prism measuring 500 mm × 100 mm × 100 mm. The test section had a length of 200 mm, a width of 100 mm, and a thickness of 50 mm, mirroring the thickness of commonly utilized R-UHPC protective and reinforcement layers. The rebars were centrally positioned, ensuring a consistent 30 mm spacing between the axes of the rebar. To achieve the intended goals, 10 sets of specimens were designed, as shown in Table 3. These encompassed five reinforcement ratios (0%, 1.7%, 3.0%, 4.7%, and 6.8%) and two fiber volume fractions (0.5% and 1.0%). It is worth noting that, while 6.8% may appear to be an exceedingly high reinforcement ratio, when one considers the entire structure instead of the UHPC protective layer exclusively (as depicted in Figure 1), this ratio remains within a reasonable range. Three specimens were produced for each set, adhering to the detailed manufacturing procedures referenced in Ref. [42].

### 2.3. Test Setup

**Figure 4:**
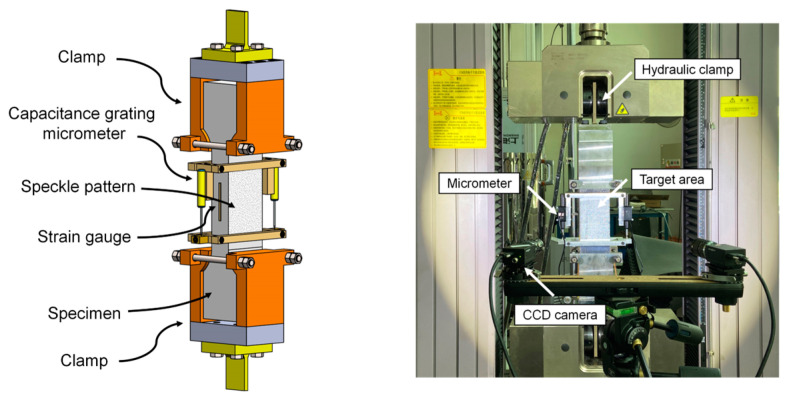
Direct tensile test setup.

As shown in Figure 4, a loading system with both ends fixed is employed to simulate actual end boundary conditions encountered in engineering practice. To minimize the difficulty in aligning the specimens, the loading clamps and specimens were processed with high precision—the clamp processing, mold making, and specimen grinding were all performed on computerized numerical control machines. The tests were performed using the displacement-controlled loading method, and the deformation rate was set at 0.1 mm/min according to Ref. [43]. It has been substantiated that the actual response of UHPC under static loading can be obtained at this deformation rate.

**Figure 5:**
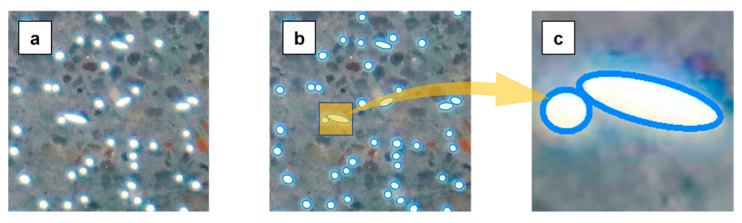
Fiber recognition: (**a**) original image; (**b**) outcome image; (**c**) local zoom.

A digital image correlation (DIC) system with an in-plane displacement resolution of up to 0.75 μm was used to capture the tensile performance of the specimen. Two micrometers were positioned on either side of the specimen to oversee its deformation and to corroborate the results obtained from DIC. Furthermore, to evaluate the effect of the rebar layouts on the internal fiber distribution features of the specimen, the image recognition method was adopted to obtain the fiber feature information inside the specimen; the recognition results are shown in Figure 5. It should be noted that the images used for image recognition are cross-sections of the specimens, which are obtained by cutting on a machine tool. To ensure that the data obtained is representative, fiber information from at least three cross-sections of specimens with the same parameters is collected. More details of the loading system, measurement equipment, and image recognition are elaborated in Ref. [42].

## 3. Local Fiber Distribution

**Figure 6:**
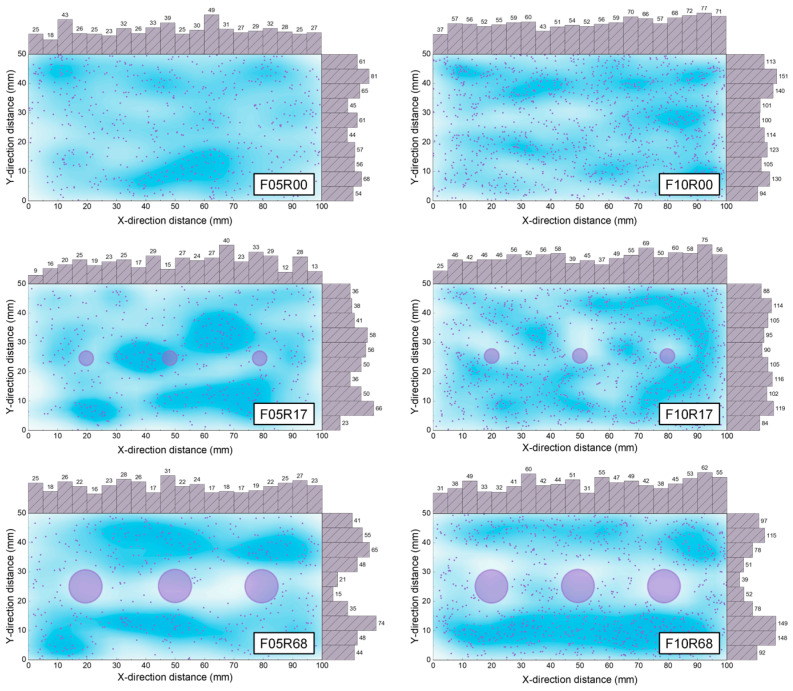
Fiber dispersion in the cross-section of the specimen.

Figure 6 displays the location and dispersion characteristics of steel fibers within the UHPC. In the unreinforced UHPC, the fibers are relatively uniformly dispersed. However, the inclusion of rebars notably compromises the uniformity of fiber dispersion, a change likely attributable to the interruption of fiber flow caused by the rebars. This phenomenon is observed as fibers predominantly clustering on one side of the rebar, and an almost complete absence of fibers on the opposing side. Furthermore, the layout of the rebars exerts a significant impact on fiber dispersion characteristics. When comparing the fiber distribution traits of specimens with a reinforcement ratio of 1.7% (with 6 mm rebar diameter and 24 mm clear rebar spacing) and a reinforcement ratio of 6.8% (with 12 mm rebar diameter and 18 mm clear rebar spacing), it becomes apparent that the inter-rebar area of the specimens with smaller clear rebar spacing is virtually devoid of fibers. This occurs because the steel fibers, with a length of up to 16 mm, find it challenging to flow into the narrow inter-rebar area (as seen in specimen F05R68). Conversely, the fiber density in the inter-rebar area of the specimens with larger clear rebar spacing is almost indistinguishable from other areas, and even a phenomenon where the fiber density in the inter-rebar area exceeds other areas due to rebar-induced obstruction of fiber flow (as seen in specimen F05R17) can occur. This observation suggests that, for a given fiber volume fraction, choosing a larger rebar diameter coupled with a smaller clear spacing when setting the rebar may increase the fiber density in regions outside the inter-rebar area, while avoiding fiber aggregation near the rebars.

**Figure 7:**
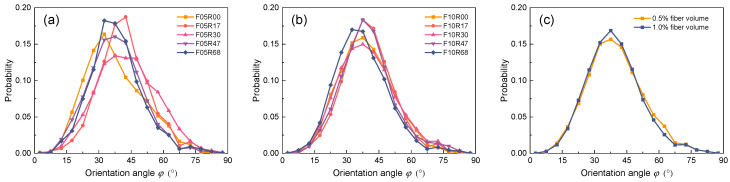
Distribution of fiber orientation angle: (**a**) fiber volume fraction of 0.5%; (**b**) fiber volume fraction of 1.0%; (**c**) comparison at two fiber volume fraction levels.

Fiber orientation significantly impacts the mechanical properties of UHPC [18,44]. Figure 7 depicts the orientation angles of the fibers within the UHPC cross-section, where the orientation angle is defined as the angle between the fiber axis and the normal to UHPC cross-section. Comparing Figure 7a,b, it is evident that the consistency of fiber orientation in UHPC with 0.5% fiber volume fraction is much worse than that in specimens with 1.0% fiber volume fraction. This difference arises primarily because the interaction among the fibers is weaker when the fiber density is lower, leading to greater randomness in the flow of the fibers within the matrix [45,46]. Meanwhile, no significant correlation appears between the presence or absence of rebars and the variation of reinforcement ratio with the fiber orientation angle distribution characteristics. This observation suggests that the disparities in fiber orientation angle distribution among the specimens may be attributable to random errors. This hypothesis gains partial support from the findings depicted in Figure 7c, where the average fiber orientation angle distribution between the two fiber volume fractions of UHPC demonstrates negligible disparity.

## 4. Mechanical Properties

### 4.1. Failure Modes

**Figure 8:**
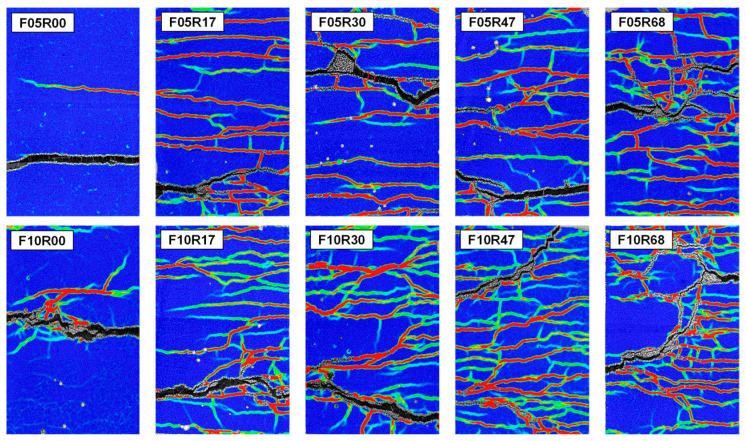
Failure modes of the specimens.

As demonstrated in Figure 8, the UHPC exhibited completely different failure modes before and after rebar reinforcement for the same fiber volume fraction, the non-blue stripe in the figure shows the location of the crack. At a fiber volume fraction of 0.5%, UHPC experiences fracture predominantly at a single surface, with minimal occurrence of multi-cracks on the flanks of the fracture surface. This finding indicates that the bridging effect provided by the steel fibers at the fracture surface at this fiber volume fraction is insufficient to induce multi-cracking behavior. Upon elevating the fiber volume fraction to 1.0%, while UHPC continues to fracture predominantly on a single surface, the bolstered bridging effect facilitated by the fibers leads to the emergence of several multi-cracks on the flanks of the fracture surface. Upon reinforcement with rebars, the failure mode of UHPC remains a single surface fracture, but as the reinforcement ratio increases, the phenomenon of secondary fracture surface initiating outside the main fracture surface becomes more evident. For example, in the specimens with a reinforcement ratio of 6.8% (F05R68 and F10R68), fracture surfaces initiate on the left and right sides of the specimen, respectively, and gradually develop through with increasing strain. In the specimens with a reinforcement ratio of 4.7% (F05R47 and F10R47), the development of secondary fracture surfaces outside the main fracture surfaces is also more apparent. From the perspective of restricting the maximum crack width propagation, the occurrence of secondary fracture surfaces is beneficial.

The addition of rebars gives UHPC with low fiber volume fraction excellent multi-cracking behaviors. At 0.5% fiber volume fraction, the UHPC without rebar was damaged with only one main crack, and almost no multi-cracks initiated except the main crack. However, with the addition of rebars, the UHPC with 0.5% fiber volume fraction had multi-cracks initiated in the whole gauge length. Similarly, for UHPC with a 1.0% fiber volume fraction without rebar, multi-cracks are initiated in a certain range on either side of the main crack. However, with the introduction of the rebar, multi-crack initiation expanded to encompass the entire gauge length. This is due to the rebar acting as “giant fibers”, providing an additional bridging effect and preventing massive pull-out of fibers at the moment of UHPC cracking. This shift in cracking pattern is only related to whether rebar is employed and shows no significant correlation with either reinforcement ratio or fiber volume fraction. Upon the fiber volume fraction being elevated from 0.5% to 1.0%, a marginal increase in crack density is observed. Furthermore, it can be noted that the number of cracks in proximity to the fracture surface in some specimens is less than in other areas, as exemplified in F10R17 and F10R68. This occurrence is primarily because the position where the fracture section is initiated tends to have poor fiber distribution quality. As a result, this location fails to induce multi-cracking behavior. Moreover, under the influence of casting-induced non-uniform fiber dispersion on the left and right sides of the specimen, as exhibited in Figure 6, the fracture surface initiates more cracks on the side with a greater concentration of fibers.

### 4.2. Load–Displacement Response

**Figure 9:**
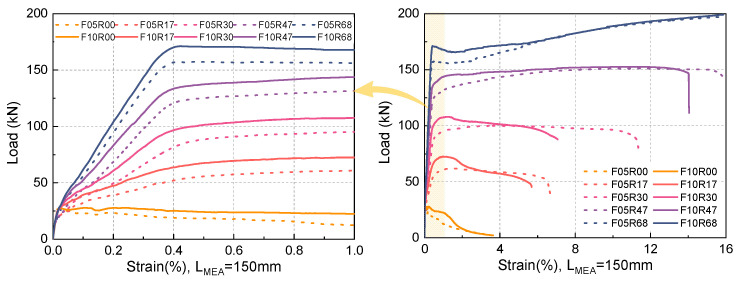
Load–strain curves of the specimens.

The load–displacement curves of all specimens are depicted in Figure 9. Both UHPC with 0.5% and 1.0% fiber volume fractions are strain-softening materials. For UHPC with a 0.5% fiber volume fraction, the load-bearing capacity decreases rapidly after initial cracking as the strain increases. For UHPC with a 0.5% fiber volume fraction, the load-bearing capacity experiences a steep decline after initial cracking as strain increases. This phenomenon can be attributed to the inadequate bridging effect facilitated by the fibers at the cracking surface, which fails to induce multi-cracking behavior and subsequently leads to an increase in the number of pulled-out fibers with an escalating strain. On increasing the fiber volume fraction to 1.0%, the enhanced bridging effect results in a slower unloading rate. However, this modest enhancement in the bridging effect is insufficient to trigger strain hardening, causing the load-bearing capacity of the UHPC to remain unchanged. It is worth noting that without the addition of rebars, elevating the fiber volume fraction from 0.5% to 1.0% yields virtually no enhancement in the load-bearing capacity. Conversely, with the addition of rebars, this same modification results in a significant rise in the load-bearing capacity. This could be attributed to the role of reinforcement in preventing the large-scale pull-out of fracture surface fibers following the initiation of cracks, thereby allowing these fibers to continue sharing the load with the rebars post-cracking. It is evident that the incorporation of rebars enables the fibers in UHPC with low fiber volume fraction to optimally contribute to the tensile load-bearing capacity.

As shown in Figure 2, the deformability of the rebars used in the R-UHPC specimens with reinforcement ratios of 1.7%, 3.0%, and 4.7% are basically the same, and the elongation at break is around 16%, but the ductility of the corresponding R-UHPC specimens varies greatly, which suggests that the difference in ductility of the R-UHPC specimens is not due to the difference in the mechanical properties of the rebars. As presented in Figure 9, the ductility of R-UHPC augments in tandem with an increasing reinforcement ratio. This can be explained by the fact that R-UHPC with a higher reinforcement ratio is more heavily influenced by the rebars in its mechanical response, thus displaying a mechanical response more akin to that of rebars. At low reinforcement ratios, the mechanical response of R-UHPC tends to emulate that of unreinforced UHPC, and its deformation is likely to concentrate near the fracture surface, leading to premature failure of the specimen. Meanwhile, an increase in fiber volume fraction contributes to a degradation in the ductility of R-UHPC. This can be attributed to the fact that the superior performing UHPC components have a more pronounced impact on the overall mechanical response of R-UHPC, aligning the mechanical response of R-UHPC more closely with that of unreinforced UHPC. When the reinforcement ratio increases to 4.7% and 6.8%, UHPC with 0.5% and 1.0% fiber volume fraction shows competitive deformability comparable to bare rebar.

**Figure 10:**
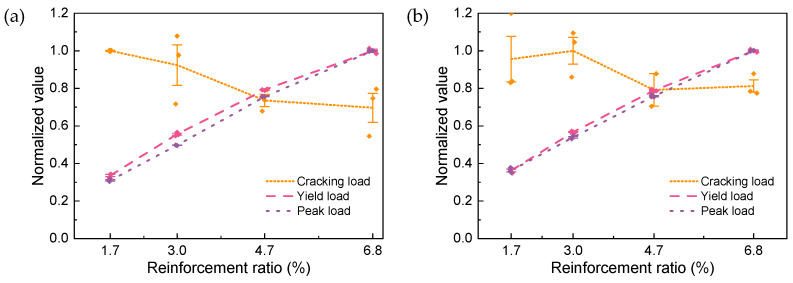
Effect of reinforcement ratio on R-UHPC feature load: (**a**) 0.5% fiber volume fraction; (**b**) 1.0% fiber volume fraction.

**Figure 11:**
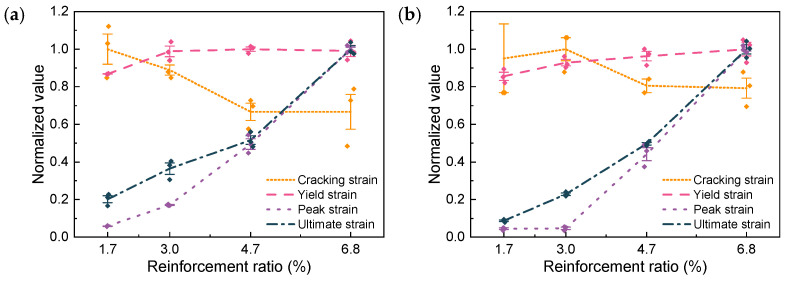
Effect of reinforcement ratio on R-UHPC feature strain: (**a**) 0.5% fiber volume fraction; (**b**) 1.0% fiber volume fraction.

Figure 10 and Figure 11 delve further into the impact of varying the reinforcement ratio on the mechanical properties of R-UHPC. The yielding point is determined by R. Park’s method [47]. All feature loads and strains are normalized. For instance, in R-UHPC with a fiber volume fraction of 0.5%, the specimen with a reinforcement ratio of 6.8% boasts the maximum average peak load, so the normalized benchmark of the peak load for all R-UHPC with a fiber volume fraction of 0.5% is defined as this average peak load. The data before normalization are shown in Table 4 and Table 5. As shown in Figure 10, the cracking load of R-UHPC generally diminishes in response to an increased reinforcement ratio. This is attributable to a larger reinforcement ratio yielding a more substantial inhibitory effect on UHPC autogenous shrinkage, resulting in amplified tensile stress being introduced into the UHPC before testing [48]. Since the same UHPC is used, both the yield and peak loads of R-UHPC approximately linearly escalate with the increase in the reinforcement ratio.

The cracking strain, akin to the cracking load, also decreases with an increasing reinforcement ratio, as shown in Figure 11. The yielding strain of R-UHPC generally augments with an increasing reinforcement ratio, although this is not an absolute trend. At a 0.5% fiber volume fraction, the yielding strain of R-UHPC only marginally increases when the reinforcement ratio progresses from 1.7% to 3.0%, subsequently exhibiting virtually no further alteration in relation to the reinforcement ratio. This phenomenon can be attributed to the higher propensity of the rebars in UHPC to experience localized deformation at the fracture surface at smaller reinforcement ratios. The extent of this localized deformation correlates with the ratio between the fiber volume fraction and the reinforcement ratio, and the effects of deformation compatibility and fiber volume fraction on rebar bonding and stress development should also be considered. This may also elucidate why, when the fiber volume fraction elevates to 1.0%, the yielding strain of UHPC consistently rises with the reinforcement ratio, although the rate of increase gradually decelerates.

The impact of the reinforcement ratio on the peak and ultimate strains is remarkably pronounced, both nearly exhibiting a linear increase with the ascending reinforcement ratio. With a 0.5% fiber volume fraction, in comparison to R-UHPC with a 1.7% reinforcement ratio, the peak strain of R-UHPC with reinforcement ratios of 3.0%, 4.7%, and 6.8% rises to 2.9, 8.5, and 17.1 times, respectively, while the ultimate strain augments to 1.8, 2.6, and 5.0 times, respectively. When the fiber volume fraction increases to 1.0%, compared to R-UHPC with a reinforcement ratio of 1.7%, the peak strain of UHPC with reinforcement ratios of 3.0%, 4.7%, and 6.8% reaches 1.0, 9.8, and 22.2 times, respectively, and the ultimate strain reaches 2.6, 5.7, and 11.5 times, respectively. A noteworthy observation is that for R-UHPC with a 1.0% fiber volume fraction, the yield strain hardly increased when the reinforcement ratio elevated from 1.7% to 3.0%, while the same operation effectively improves the yield strain of R-UHPC with a 0.5% fiber volume fraction. This phenomenon further attests to the positive correlation between the degree of localized deformation of the rebars and the fiber volume fraction.

**Figure 12:**
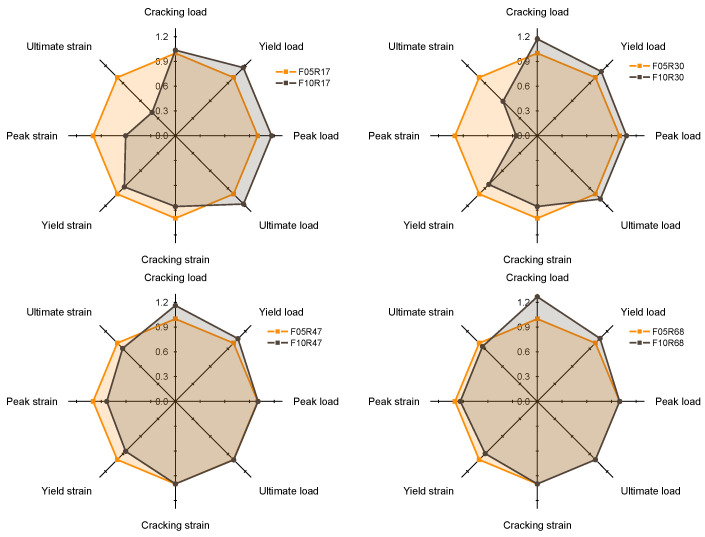
Effect of fiber volume fraction on R-UHPC tensile performance.

To evaluate the mechanical property change induced by fiber volume fraction variation, Figure 12 compares the mechanical feature points when the fiber volume fraction is 0.5% and 1.0% under each reinforcement ratio. Broadly, reducing the fiber volume fraction lessens the load-bearing capacity of R-UHPC but facilitates an enhancement in ductility. This transformation in mechanical properties, brought on by alterations in fiber volume fraction, is influenced by the reinforcement ratio—the larger the reinforcement ratio, the more marginal the change in R-UHPC properties prompted by modifications in the fiber volume fraction. The increase in cracking load with increasing fiber volume fraction can be attributed to a decrease in free shrinkage of the UHPC [49,50].

The peak and ultimate loads escalate with an increase in fiber volume fraction at lower reinforcement ratios (1.7% and 3.0%), but they remain virtually unchanged with fiber volume fraction at higher reinforcement ratios (4.7% and 6.8%). This observation arises from the fact that R-UHPC with a higher reinforcement ratio is a strain-hardening material (as illustrated in Figure 9), and when R-UHPC reaches the peak and ultimate load, the fracture surface of the specimen has undergone substantial deformation. For R-UHPC, which is a composite material of UHPC and steel rebars, when the deformation of the specimen is large and mainly concentrated in the main crack, the UHPC at the main crack will no longer be able to share the load with the rebars because of many steel fibers being pulled out. Under these circumstances, the UHPC and rebar components no longer have the co-working effect that the composite material has, leading to the peak and ultimate load-bearing capacity of R-UHPC being completely determined by the property of the rebar.

The substantial effect of modifying the fiber volume fraction on the deformability of R-UHPC is of greater interest than the characteristic load. For R-UHPC with reinforcement ratios of 1.7% and 3.0%, elevating the fiber volume fraction from 0.5% to 1.0% results in a marked decrease in its ductility. More specifically, the yield, peak, and ultimate strains of R-UHPC with a reinforcement ratio of 1.7% diminish by 12.4%, 39.5%, and 60.0%, respectively, while those with a reinforcement ratio of 3.0% recede by 16.6%, 74.2%, and 41.0%, respectively. However, when the reinforcement ratio escalates to 4.7% and 6.0%, the adverse impact of increasing the fiber volume fraction on the tensile ductility of R-UHPC is mitigated. The yield, peak, and ultimate strain of R-UHPC with a reinforcement ratio of 4.7% decrease by 14.5%, 16.7%, and 9.4%, respectively, while those with a reinforcement ratio of 6.0% recede by 10.5%, 6.6%, and 5.9%, respectively. This pattern suggests that when the reinforcement ratio is low, even a modest increase in fiber volume fraction can lead to severe degradation in deformability. If a low reinforcement ratio is imperative, the fiber volume fraction should be rigorously restricted to a lower level, and increasing the reinforcement ratio can counteract the negative influence of escalating fiber volume fraction on tensile ductility.

To underscore, an analysis combining Figure 9 and Figure 12 indicates that while decreasing the fiber volume fraction is seen as a viable option to enhance the ductility of R-UHPC—and indeed it does serve this purpose—when the fiber volume fraction drops to as low as 1.0%, or even 0.5%, the ductility of R-UHPC with a lower reinforcement ratio (such as 1.7%) still substantially deteriorates in comparison to bare steel rebars. This condition is mitigated when the reinforcement ratio is escalated to 3.0%. Thus, if feasible, it is advisable to utilize UHPC with a low fiber volume fraction at a high reinforcement ratio with the objective of achieving exceptional ductility.

### 4.3. Load-Bearing Capacity

Research surrounding the mechanical properties of R-UHPC is still not comprehensive, and as such, the method for calculating its tensile load-bearing capacity is currently under exploration. A few researchers have pioneered methods for estimating the load-bearing capacity of R-UHPC, and have demonstrated their reliability within certain parameters [27]:

(1) $$N_{u}=f_{ct}A_{c}+f_{y}A_{s}$$

where $N_{u}$ is the load-bearing capacity of the R-UHPC, $f_{ct}$ and $A_{c}$ are the tensile strength and area of the UHPC, respectively, and $f_{y}$ and $A_{s}$ are the yield strength and area of the rebars, respectively. This calculation method, which directly combines the tensile load-bearing capacity of the UHPC component with the yield load-bearing capacity of the rebars, operates under the assumption that the steel rebar component yields precisely when the UHPC component reaches its bearing capacity. However, this condition is not universally applicable.

**Figure 13:**
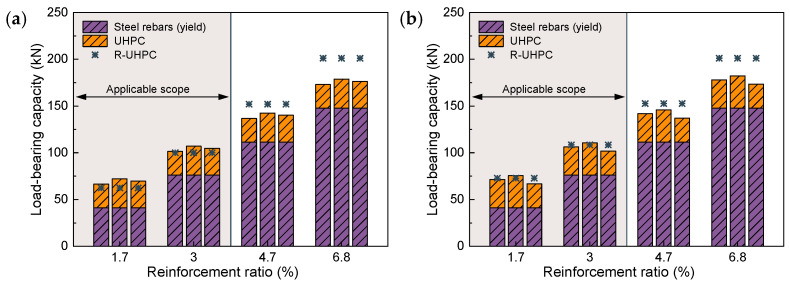
Calculation of the load-bearing capacity of R-UHPC based on the yield strength of steel rebar: (**a**) fiber volume fraction of 0.5%; (**b**) fiber volume fraction of 1.0%.

Figure 13 compares the load-bearing capacity of R-UHPC calculated via Equation (1) with the actual tensile load-bearing capacity. While Equation (1) accurately predicts the load-bearing capacity of R-UHPC with a lower reinforcement ratio, the error escalates rapidly when applied to R-UHPC with a higher reinforcement ratio. This divergence is largely attributed to the discrepancy between the actual loading state of the R-UHPC and the foundational assumptions of Equation (1) when R-UHPC reaches its tensile load-bearing capacity. In instances where the reinforcement ratio is high, it is observed that the rebars remain in the strain-hardening stage even after a substantial number of fibers have been pulled out. In such scenarios, load-bearing capacity predictions derived from the yield strength of the rebars tend to underestimate the actual load-bearing capacity of the R-UHPC.

Essentially, the load-bearing capacity of R-UHPC, encompassing both the UHPC and rebar components, can be calculated according to the superposition principle using the following equation:

(2) $$N_{u}=f_{ct,a}A_{c}+f_{s,a}A_{s}$$

where $f_{ct,a}$ and $f_{s,a}$ are the actual tensile stresses of the UHPC component and rebar component when the R-UHPC reaches tensile load-bearing capacity, respectively, and $A_{c}$ and $A_{s}$ are the areas of the UHPC component and rebar component, respectively.

Equation (1) can be considered as a special case of Equation (2), applicable to R-UHPC where the UHPC component reaches load-bearing capacity when the rebar component just yields, at which time $f_{ct,a}$ and $f_{s,a}$ can be respectively taken as $f_{ct}$ and $f_{y}$. Drawing from the preceding analysis, the rebars in R-UHPC with lower reinforcement ratios experience considerable localized deformation at the main crack, and these rebars tend to have yielded by the time the UHPC component reaches its load-bearing capacity. Consequently, Equation (1) displays high accuracy in such situations. Moreover, when the reinforcement ratio is low or the fiber volume fraction is high, the deformation of rebars at the main crack is markedly concentrated. There may be instances where the rebars at the main crack have already yielded and transitioned into the strain-hardening stage, but the actual load borne by the steel fibers at the main crack falls short of its load-bearing capacity because the crack has not fully opened. In these circumstances, Equation (1) may mitigate the discrepancy between the predicted and actual load-bearing capacity by overestimating the load-bearing capacity contribution of the UHPC component and underestimating that of the rebar component. This phenomenon, in which a good prediction outcome is obtained despite the foundational assumptions of Equation (1) being inconsistent with the actual mechanical failure mechanism, needs to be given extra attention to avoid its misuse for structural calculations.

When the R-UHPC with a higher reinforcement ratio reaches its load-bearing capacity, the tensile stress $f_{ct,a}$ of the UHPC component at the fracture surface can be approximated as zero due to a large amount of fiber being pulled out. In this scenario, the rebar is the main source of load-bearing capacity, so $f_{s,a}$ can be taken as the ultimate strength $f_{u}$ of the rebar. Hence, Equation (2) can be simplified to:

(3) $$N_{u}=f_{u}A_{s}$$

**Figure 14:**
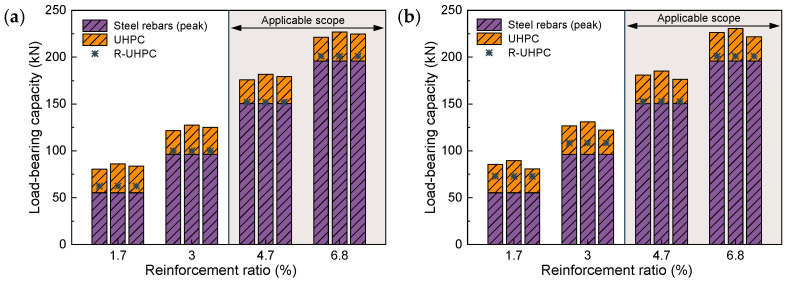
Calculation of the load-bearing capacity of R-UHPC based on the peak strength of steel rebar: (**a**) fiber volume fraction of 0.5%; (**b**) fiber volume fraction of 1.0%.

Figure 14 illustrates the comparison between the predicted and actual load-bearing capacity of R-UHPC following Equation (3). This calculation method accurately predicts the load-bearing capacity of R-UHPC with reinforcement ratios of 4.7% and 6.8%, in contrast to Equation (1), which significantly underestimates the load-bearing capacity. Notably, an aforementioned coincidence arises once again: even though the foundational assumption does not align with the actual mechanical failure mechanism, the load-bearing capacity predicted following Equation (3) demonstrates commendable consistency with the actual load-bearing capacity of R-UHPC with a fiber volume fraction of 0.5%. As anticipated, this fortuitous match fails as the fiber volume fraction escalates to 1.0%.

During the cracking process, R-UHPC experiences a complex and recurring redistribution of load between its UHPC and rebar components, which is influenced by the ratio of fiber volume fraction to reinforcement ratio [42]. In circumstances where the reinforcement ratio is low, the additional load transferred to the rebars due to the cracking of the UHPC component could potentially cause the rebars at the crack to yield. Conversely, when the reinforcement ratio is high, a scenario might emerge where all fibers at the fracture surface have been pulled out, yet the rebars at the fracture surface remain in the strength hardening stage. Generally, the method for calculating the load-bearing capacity of R-UHPC should be determined in accordance with its actual mechanical behavior and corresponding mechanism, or more precisely, according to the ratio of its fiber volume fraction and reinforcement ratio. Given varying fiber volume fractions and reinforcement ratios, the following situations could manifest:

1. The rebar component, due to excessive localized deformation, enters the yielding stage prior to the UHPC component reaching its load-bearing capacity.
2. Upon the UHPC component reaching its load-bearing capacity, the rebar component enters the strength hardening stage, or just reaches ultimate strength, or enters the necking stage.
3. Both the UHPC component and the rebar component concurrently reach their load-bearing capacities.
4. When the load-bearing capacity of the UHPC component declines due to many fibers being pulled out, the rebar component continues in the strain-hardening stage.

Evidently, to propose a universally applicable calculation method for the load-bearing capacity of R-UHPC, it is necessary to clarify the loading stage of the UHPC component and rebar component when R-UHPC reaches its load-bearing capacity under different combinations of reinforcement ratios and fiber volume fractions. This endeavor calls for an extensive number of experiments along with comprehensive parameter discussions.

Drawing from the existing literature and the test results of this paper, the load-bearing capacity of R-UHPC calculated in line with Situation 1 (severe localized deformation of rebar exists, Equation (1)) and Situation 4 (the localized deformation of the rebars is not pronounced, Equation (3)) appears to be fairly representative. The load-bearing capacities of the remaining situations can be calculated by applying correction coefficients to those of Situations 1 and 4, which would be deemed adequate for engineering practice. However, it remains imperative to conduct more comprehensive investigations to propose a predictive method for the load-bearing capacity of R-UHPC that considers varying combinations of fiber volume fraction and reinforcement ratios.

## 5. Cracking Behavior

### 5.1. Cracking Process

To quantitatively characterize crack features, a method of collecting crack data at a fixed location was used. The collecting locations were determined to be at the positions of two vertical trisection lines within the measurement area of the specimen. This placement not only avoids the edges of the specimen but also maintains an appropriate distance, aiming to achieve an objective and comprehensive collection of crack features.

**Figure 15:**
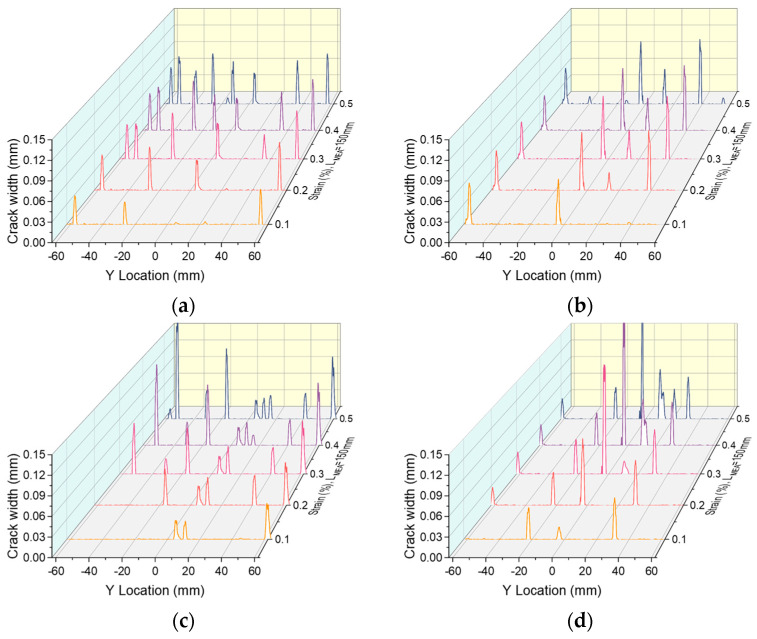
Cracking process in R-UHPC with 0.5% fiber volume fraction: (**a**) *ρ* = 1.7%; (**b**) *ρ* = 3.0%; (**c**) *ρ* = 4.7%; (**d**) *ρ* = 6.8%.

**Figure 16:**
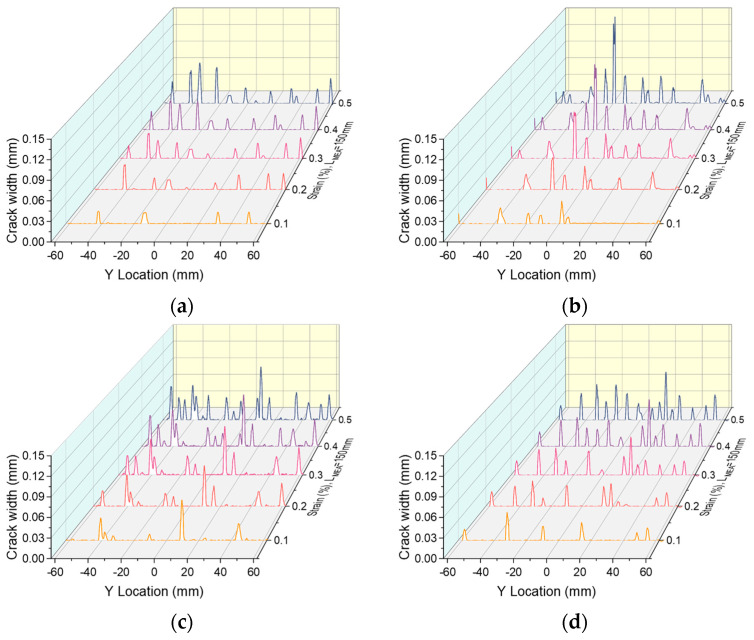
Cracking process in R-UHPC with 1.0% fiber volume fraction: (**a**) *ρ* = 1.7%; (**b**) *ρ* = 3.0%; (**c**) *ρ* = 4.7%; (**d**) *ρ* = 6.8%.

Figure 15 and Figure 16 present the cracking process with increasing strain for R-UHPC with 0.5% and 1.0% fiber volume fractions, respectively. Alterations to the fiber volume fraction and reinforcement ratio do not significantly influence the cracking pattern of R-UHPC. In all specimens, crack development is synchronous across the entire gauge length as strain escalates, with both the number and width of the cracks expanding in relation to the strain increase. In conjunction with Figure 8, it is evident that the integration of rebars can bestow upon UHPC with low fiber volume fraction exceptional multi-cracking behavior. Referring to Figure 15a,b and Figure 16a,b, it becomes clear that the degree of deformation concentration in R-UHPC with a fiber volume fraction of 1.0% is notably higher than that in R-UHPC with a fiber volume fraction of 0.5%. However, with an escalating reinforcement ratio, the strain concentration degree of R-UHPC with a fiber volume fraction of 1.0% gradually diminishes (Figure 16c,d), aligning with the previous analysis of localized deformation of reinforcement. Conversely, in R-UHPC with 0.5% fiber volume fraction, the strain concentration increases as the reinforcement ratio heightens. This phenomenon may be attributed to the fact that the uniformity of fiber dispersion in R-UHPC with a 0.5% fiber volume fraction is intrinsically inferior to that in R-UHPC with a 1.0% fiber volume fraction. Increasing the reinforcement ratio exacerbates this non-uniformity, provoking the deformation to tend to concentrate in regions with lower fiber density.

**Figure 17:**
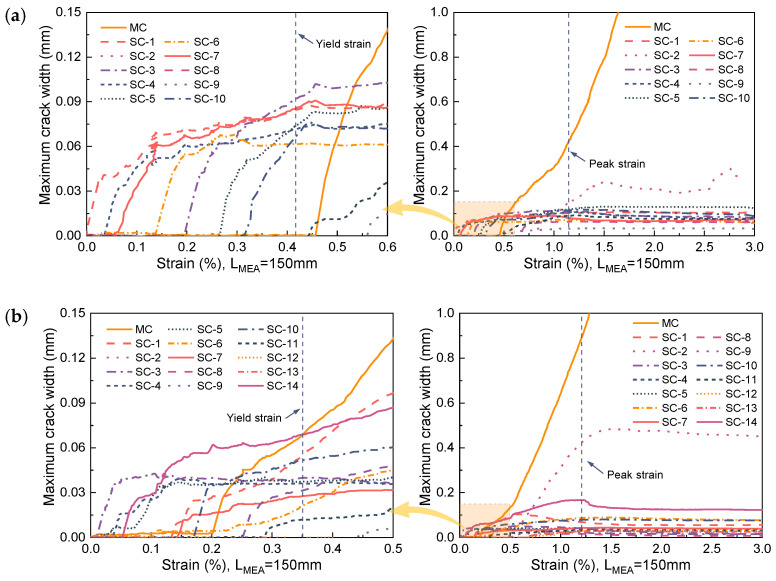
Crack width propagation in R-UHPC: (**a**) F05R17; (**b**) F10R17.

Figure 17 illustrates the typical progression of the width of all cracks on a single R-UHPC specimen in correlation with increasing strain. Broadly, the propagation of crack width in line with escalating strain aligns well with. In the instance of R-UHPC with a 0.5% fiber volume fraction (Figure 17a), as strain enlarges, the first crack appears and rapidly widens until the initiation of the second crack. Following the emergence of the second crack, it swiftly expands, while the growth of the first crack concurrently decelerates until the advent of the third crack. This sequence repeats until the rebar located at the section of a particular crack yields. Once the rebar yields, the width of the main crack (labeled as MC in Figure 17) swiftly expands, during which period, the width of the other cracks ceases to enlarge or increases only marginally with strain increment. After reaching the peak strain, the width of the cracks, apart from the main crack, diminishes with the gradual unloading of the specimen. As can be seen from Figure 17b, after the fiber volume fraction increases to 1.0%, the main crack begins to propagate rapidly before the yield of the R-UHPC, which is due to the heightened fiber volume fraction intensifying the degree of localized deformation of the rebar.

The propagation of the main crack width with strain can be segmented into two stages according to the propagation rate. During the initial stage, the rate of crack propagation is relatively sluggish, attributed to the bridging effect exerted by the steel fibers on the cracked surface. As the fibers are gradually pulled out, the speed of crack propagation in the second stage significantly accelerates. The critical strain demarcating the first and second stages is about 1.0% in F05R17, decreasing to roughly 0.5% in F10R17. This differential reflects the degree of deformation concentration in the rebars. Further, according to Figure 17, the propagation process of maximum crack width with strain can be segmented into three distinct stages: The initial stage marks the initiation of cracks, characterized by the rapid propagation of the first crack. Subsequently, the multi-crack development stage begins with the emergence of the second crack and concludes when the main crack initiates and widens significantly. Lastly, the third stage denotes the swift expansion phase of the main crack, commencing when it reaches its maximum width and continuing until the specimen fractures. 

### 5.2. Cracks Number and Spacing

**Figure 18:**
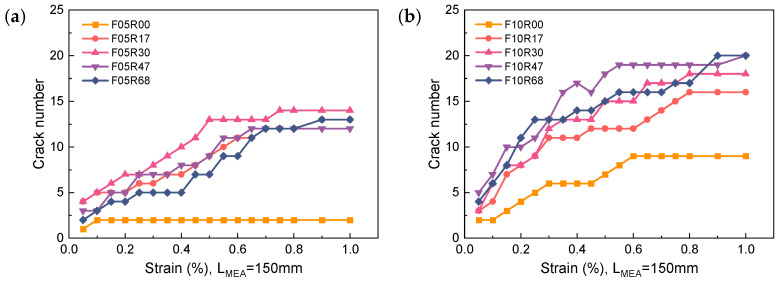
Development of crack number: (**a**) fiber volume fraction of 0.5%; (**b**) fiber volume fraction of 1.0%.

Figure 18 depicts the development of crack numbers with strain in R-UHPC. Compared to unreinforced UHPC, R-UHPC displays a markedly higher crack count due to its superior multi-cracking behavior. Under identical reinforcement ratios, an increase in fiber volume fraction can also contribute to a more pronounced multi-crack effect. R-UHPC with a fiber volume fraction of 1.0% shows a significantly higher crack count than its 0.5% fiber volume fraction counterpart. However, changes in reinforcement ratio do not significantly impact the number of cracks. 

**Figure 19:**
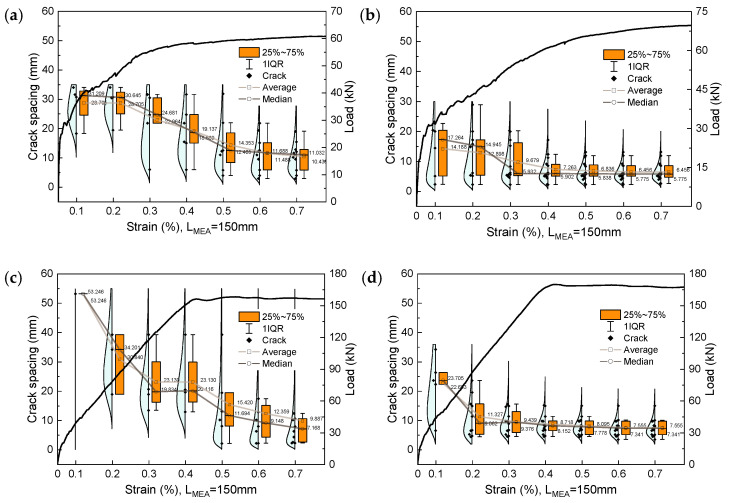
Development of crack spacing: (**a**) F05R17; (**b**) F10R17; (**c**) F05R68; (**d**) F10R68.

Figure 19 shows the development of crack spacing in R-UHPC with strain. It should be noted that crack spacing is the spacing between two neighboring cracks at the location of the crack data collection. When elevating the fiber volume fraction from 0.5% to 1.0%, the average crack spacing of the R-UHPC with a reinforcement ratio of 1.7% decreases from 10.4 mm to 5.8 mm, but similar phenomena do not occur in specimens with a reinforcement ratio of 6.8%. Also, variations in reinforcement ratio exert inconsistent effects on crack spacing depending on the fiber volume fraction. For instance, raising the reinforcement ratio can decrease crack spacing when the fiber volume fraction is 0.5% (Figure 19a,c), but it increases crack spacing when the fiber volume fraction is elevated to 1.0% (Figure 19b,d). The above analysis of crack spacing does not support the formation of noteworthy conclusions, which may be due to the crack spacing being influenced by multiple factors and not solely determined by fiber volume fraction and reinforcement ratio.

### 5.3. Maximum Crack Width

**Figure 20:**
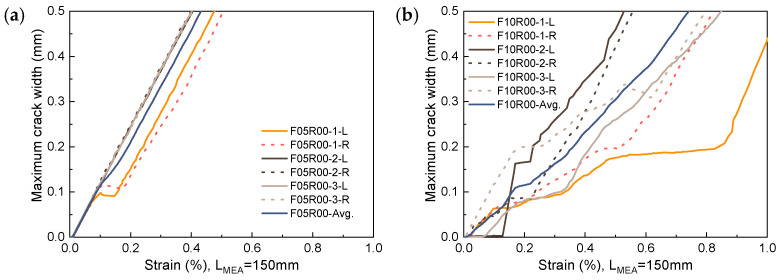
Propagation of maximum crack width in UHPC: (**a**) F05R00; (**b**) F10R00.

The propagation of the maximum crack width with strain for the unreinforced UHPC is shown in Figure 20. To ensure data representativeness, the maximum crack width data is extracted from the trisection lines on both sides of each specimen. In Figure 20, the label F05R00-1-L refers to data extracted from the left trisection line of specimen F05R00-1. For unreinforced UHPC with a fiber volume fraction of 0.5% or lower, once a crack initiates, its propagation progresses rapidly with width increasing linearly alongside strain increments. In this case, fibers offer minimal restriction to crack propagation, as evidenced by the observed failure mode, where hardly any multi-cracking occurs on either side of the fracture surface. When the fiber volume fraction elevates to 1.0%, the propagation of the main crack with increasing strain is inhibited by the fibers, inducing multi-cracking behavior within a local range on both sides of the fracture surface, as shown in Figure 6. Notwithstanding, due to the varying uniformity of fiber dispersion and fiber orientation, this inhibitory effect is not stable. This instability leads to a high level of dispersion in the progression of the maximum crack width with increasing strain, as exhibited in Figure 20b. Consequently, this poses challenges in accurately predicting the maximum crack width.

**Figure 21:**
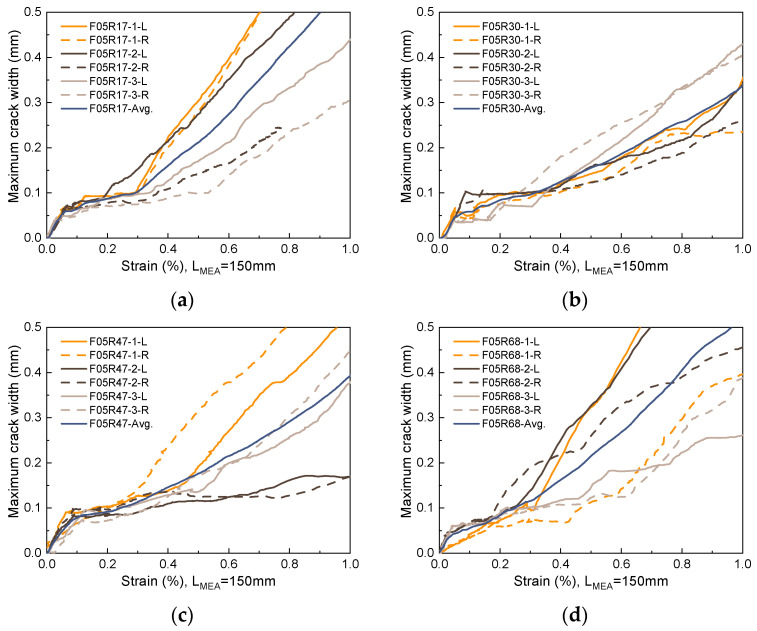
Propagation of maximum crack width in R-UHPC with 0.5% fiber volume fraction: (**a**) *ρ* = 1.7%; (**b**) *ρ* = 3.0%; (**c**) *ρ* = 4.7%; (**d**) *ρ* = 6.8%.

**Figure 22:**
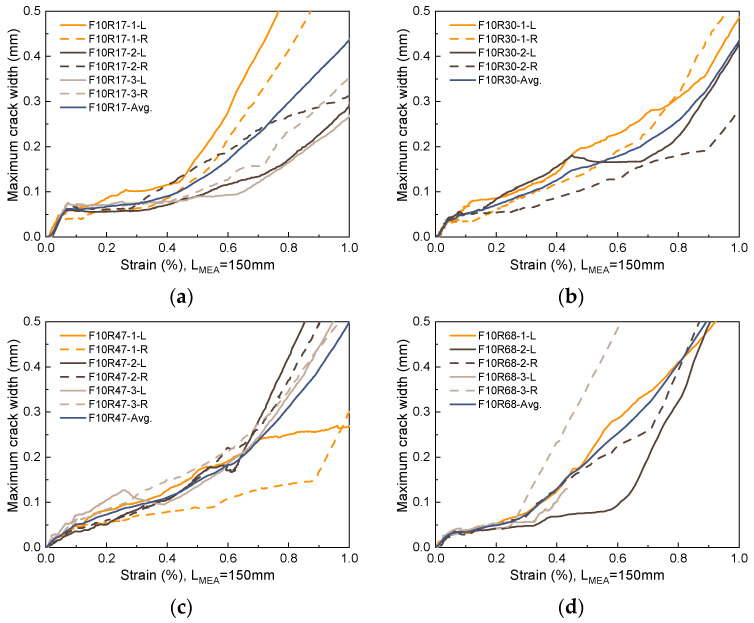
Propagation of maximum crack width in R-UHPC with 1.0% fiber volume fraction: (**a**) *ρ* = 1.7%; (**b**) *ρ* = 3.0%; (**c**) *ρ* = 4.7%; (**d**) *ρ* = 6.8%.

Figure 21 and Figure 22 show the propagation of the maximum crack width with strain for R-UHPC with 0.5% and 1.0% fiber volume fractions, respectively. Combined with Figure 20, it can be seen that with the addition of rebars, the ability of UHPC to restrict the propagation of maximum crack width is significantly enhanced. This improvement is attributable to the additional bridging effect brought about by the rebars, resulting in superior dispersion of cracks. However, post-yielding, dispersion in the propagation of the maximum crack width with strain begins to increase. This is primarily due to a shift in the principal source of strain growth after yield, transitioning from an increase in crack numbers to an expansion of the main crack width. Furthermore, the cross-section where the main crack is situated typically exhibits the poorest global fiber distribution quality, and the variation in global worst fiber distribution quality across different specimens is significant. The incorporation of rebar has improved the consistency of UHPC cracking behavior, providing the possibility to accurately predict its maximum crack width, which is of great engineering significance.

**Figure 23:**
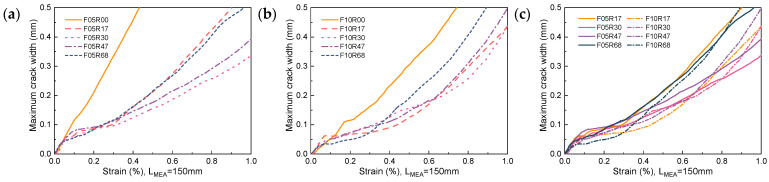
Propagation of average maximum crack width: (**a**) fiber volume fraction of 0.5%; (**b**) fiber volume fraction of 1.0%; (**c**) comparison at two fiber volume fraction levels.

Figure 23 compares the ability of different reinforcement ratios and fiber volume fractions for R-UHPC to restrict maximum crack width. The figure reveals that incorporating rebar significantly enhances the ability of UHPC with low fiber volume fraction to restrict maximum crack width, particularly noticeable in UHPC with a 0.5% fiber volume fraction. For instance, when setting the maximum crack width limit to 0.2 mm, 0.3 mm, and 0.4 mm, the strain of R-UHPC with a 0.5% fiber volume fraction experiences an increase of at least 145.5%, 131.5%, and 117.9% following reinforcement, respectively. Concurrently, the strain of R-UHPC with a 1.0% fiber volume fraction escalates by a minimum of 43.5%, 36.0%, and 25.7% after reinforcement, respectively.

Compared to the significant impact of whether rebars are provided on the effectiveness of UHPC to restrict the maximum crack width, alterations in the reinforcement ratio led to little change. Meanwhile, the effect of changing the reinforcement ratio on the effectiveness of R-UHPC to restrict the maximum crack width is not uniform, with neither too low nor too high values being ideal. As illustrated in Figure 23c, due to the better bridging effect, an elevation in fiber volume fraction can enhance the efficacy of UHPC to some extent in restricting the maximum crack width, especially within the low strain stage. With the goal of restricting the propagation of the maximum crack width, for R-UHPC with a fiber volume fraction of 0.5%, reinforcement ratios of 3.0% and 4.7% are deemed most suitable, while for R-UHPC with a fiber volume fraction of 1.0%, all reinforcement ratios except 6.8% are suitable.

### 5.4. Fiber Efficiency in Restricting Maximum Crack Propagation

As aforementioned, the cost of steel fibers constitutes a significant portion of the overall high cost of UHPC. For the efficient use of steel fibers, determining the efficiency of steel fibers in improving UHPC performance under various parameter combinations is essential. This would guide the selection of fiber volume fraction. The efficiency $\eta_{strain}$ of steel fibers in restricting the maximum crack propagation at different strain levels can be calculated through the following equation, which is derived from a modification of the equation presented in Ref. [29]:

(4) $$\eta_{strain}\left(\varepsilon \right)=\frac{w_{s}\left(\varepsilon \right)}{w_{b}\left(\varepsilon \right)}\cdot \frac{V_{f,b}}{V_{f,s}}$$

where $w_{s}\left(\varepsilon \right)$ and $w_{b}\left(\varepsilon \right)$ are the maximum crack width at a strain of ε for the evaluated and benchmark specimen, respectively, and $V_{f,s}$ and $V_{f,b}$ are the fiber volume fractions of the evaluated and benchmark specimens, respectively.

**Figure 24:**
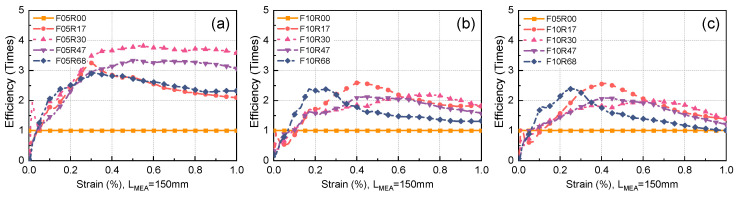
Efficiency of fiber in restricting the maximum crack width: (**a**) fiber volume fraction of 0.5%, (**b**) fiber volume fraction of 1.0%, (**c**) comparison.

Figure 24 illustrates the effectiveness of steel fibers in restricting maximum crack propagation across various strain levels. The incorporation of rebar notably enhances the capacity of UHPC to restrict the propagation of maximum crack width. Specifically, when the fiber volume fraction is set at 0.5%, the efficiency of steel fibers in restricting maximum crack propagation experiences a remarkable increase of up to 282.3% compared to the reference efficiency, where the reference specimen represents unreinforced UHPC with a fiber volume fraction of 0.5%. This corresponds to a specimen with a reinforcement ratio of 3.0%, as demonstrated in Figure 24a. When strain surpasses 0.05%, incorporating rebar can yield advantages in enhancing the efficiency of fibers in restricting the maximum crack propagation. After raising the fiber volume fraction to 1.0%, the enhancement effect of adding rebar on fiber efficiency diminishes but still achieves an increase of 159.7% compared to the reference efficiency (the reference specimen being unreinforced UHPC with a fiber volume fraction of 1.0%). This corresponds to a reinforcement ratio of 1.7%. In the range where tensile strain is greater than 0.1%, incorporating rebar can yield benefits in terms of improving fiber efficiency. The effect of changing the reinforcement ratio on improving fiber efficiency varies across different strains. For instance, UHPC with a reinforcement ratio of 6.8% exhibits a higher improvement effect on fiber efficiency in low-strain ranges compared to other reinforcement ratios, but in high-strain ranges, its improvement effect on fiber efficiency is less pronounced than other reinforcement ratios.

Figure 24c assesses the fiber efficiency in R-UHPC with a 1.0% fiber volume fraction, utilizing unreinforced UHPC with a 0.5% fiber volume fraction as the benchmark specimen. This comparative analysis enables the evaluation of the efficacy of each 0.5% fiber volume fraction increment in 1.0% fiber volume fraction UHPC in restricting maximum crack propagation. Comparing Figure 24a,c, it can be seen that each 0.5% fiber volume fraction in R-UHPC with a 1.0% fiber volume fraction is less efficient in restricting the maximum crack propagation than R-UHPC with a 0.5% fiber volume fraction. Elevating the fiber volume fraction from 0.5% to 1.0% diminishes the fiber efficiency. From the perspective of improving the efficiency of fibers in restricting the maximum crack propagation, choosing R-UHPC with a 0.5% fiber volume fraction and a 3.0% reinforcement ratio is relatively efficient. It is important to emphasize that there is no one parameter combination that gives fibers the highest efficiency across all strain levels. In practical engineering, the appropriate parameters should be determined based on the strain level at which the engineering structure is in service.

In practical engineering, different maximum crack width limits are proposed depending on the service environments of the structure, to ensure that the structure has sufficient durability. Therefore, clarifying the efficiency of fibers in restricting a specified maximum crack width in R-UHPC under diverse parameter combinations can provide a more direct reference for engineering practice. The efficiency of fibers in restricting a specified maximum crack width $\eta_{width}$ can be calculated using the following equation:

(5) $$\eta_{width}\left(w\right)=\frac{\varepsilon_{s}\left(w\right)}{\varepsilon_{b}\left(w\right)}\cdot \frac{V_{f,b}}{V_{f,s}}$$

where $\varepsilon_{s}\left(w\right)$ and $\varepsilon_{b}\left(w\right)$ are the strains of the evaluated and benchmark specimens, respectively, when the maximum crack width reaches w.

**Figure 25:**
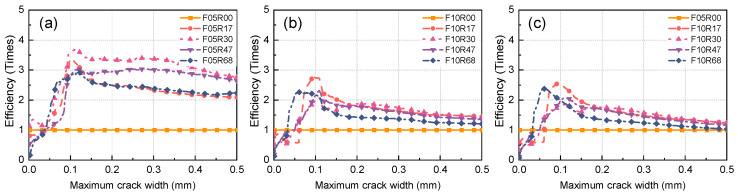
Efficiency of fiber in restricting maximum crack width: (**a**) fiber volume fraction of 0.5%, (**b**) fiber volume fraction of 1.0%, (**c**) comparison.

The efficiency of steel fibers in restricting a specified maximum crack width under varying parameter combinations is depicted in Figure 25. When the maximum crack width limit exceeds 0.05 mm, the addition of rebars can enhance fiber efficiency. The fiber efficiencies in R-UHPC with fiber volume fractions of 0.5% and 1.0% augment up to 267.0% and 175.8%, respectively, compared to the benchmark efficiency. The corresponding reinforcement ratios are 3.0% and 1.7%. Once the maximum crack width limit surpasses 0.10 mm, the benefits conferred by the inclusion of steel rebars in amplifying fiber efficiency progressively diminish. This trend emerges as the crack propagation enters its third stage, during which the source of tensile deformation gradually transitions from the elevation in crack numbers to the propagation of the main crack. The effect of altering the reinforcement ratio on fiber efficiency for restricting a specified maximum crack width does not adhere to any discernible pattern.

## 6. Conclusions

In this study, the impacts of varying the reinforcement ratio on the tensile load-bearing capacity, ductility, and cracking behavior of UHPC with a low fiber volume fraction are investigated. The efficient fiber volume fraction and reinforcement ratio combinations are recommended to meet different engineering requirements. From the direct tensile test results of 30 UHPC specimens, the following conclusions can be drawn:

1. Adopting UHPC with a low fiber volume fraction can significantly mitigate the ductility deterioration of R-UHPC, and both increasing the reinforcement ratio and decreasing the fiber volume fraction contribute to the improvement of ductility.
2. Reducing the fiber volume fraction was particularly effective in improving the ductility of R-UHPC with low reinforcement ratios; for R-UHPC with reinforcement ratios of 1.7% and 3.0%, the reduction of the fiber volume fraction from 1.0% to 0.5% resulted in an increase in peak ductility of 55.4% and 287.7%, respectively. The ductility of R-UHPC with low fiber volume fraction increases approximately linearly with increasing reinforcement ratio.
3. The failure modes of R-UHPC are determined by the ratio of reinforcement ratio and fiber volume fraction, rather than a single parameter, which also means that R-UHPC with different parameters may require different methods to predict tensile load-bearing capacity. For R-UHPC with significant ductility degradation, the load-bearing capacity can be calculated by superimposing the tensile load-bearing capacity of the UHPC component and the yielding load-bearing capacity of the rebar component. For R-UHPC with no substantial ductility degradation, the load-bearing capacity can be calculated by considering the contribution of rebars only.
4. Incorporating steel rebars into UHPC with a low fiber volume fraction can significantly improve its capacity to restrict the maximum crack propagation. With maximum crack width up to 0.20 mm, the strains experienced by R-UHPC with fiber volume fractions of 0.5% and 1.0% increased by at least 145.5% and 43.5%, respectively, compared to unreinforced UHPC. Compared to whether rebars are incorporated, varying the reinforcement ratio does not remarkably affect the ability of R-UHPC to restrict the maximum crack propagation.
5. Increasing the fiber volume fraction from 0.5% to 1.0% results in R-UHPC demonstrating better multi-cracking behavior, yet there is only a marginal enhancement in its ability to restrict maximum crack propagation. Enhancing the ability of R-UHPC to restrict the maximum crack propagation by augmenting the fiber volume fraction is inefficient.
6. If excellent ductility is essential, R-UHPC with a reinforcement ratio greater than 4.7% combined with a 0.5% fiber volume fraction can be adopted. If the capacity to restrict maximum crack propagation is primarily required, R-UHPC with a reinforcement ratio of 1.7% combined with a 0.5% fiber volume fraction is more appropriate. Increasing the fiber volume fraction from 0.5% to 1.0% at the same reinforcement ratio will yield little benefit other than increased load-bearing capacity.

This study has demonstrated at the material level that UHPC with a low fiber volume fraction has advantages in reducing material costs and improving the tensile ductility of R-UHPC, and its ability to control the maximum crack width meets the needs of most cases. However, the working performance of R-UHPC with low fiber volume fraction at the member level remains to be confirmed, as there may be differences between its performance as an independent component and its performance in composite members. Therefore, it is necessary to continue research on the performance of members using R-UHPC with a low fiber volume fraction as a reinforcement and protective layer. If the R-UHPC with a low fiber volume fraction also exhibits excellent mechanical behavior at the structural level, further research into its cost, efficiency, and carbon footprint would be necessary.

## Figures and Tables

**Table 1:** Mechanical properties of UHPC samples used in this study.

Fiber Volume Fraction (vol%)	Tensile Strength *f*_t_ (MPa)	Compressive Strength *f*_c_ (MPa)	Elastic Modulus *E* (GPa)
0.5	5.7 ± 0.6	143.9 ± 5.8	41.6 ± 1.3
1.0	6.0 ± 0.9	145.7 ± 4.8	43.3 ± 0.9

**Table 2:** Mechanical properties of steel rebar.

Diameter *d* (mm)	Elastic Modulus *E* (GPa)	Yield Strength *f*_y_ (MPa)	Ultimate Strength *f*_u_ (MPa)	Ultimate Strain *ε*_u_ (%)
6	206	483	649	11.0
8	212	504	640	9.3
10	202	473	639	11.1
12	208	434	597	13.8

**Table 3:** Experimental parameter design.

Types	Specimen	Fiber Volume Fraction V*_f_* (vol%)	Reinforcement Ratio *ρ* (%)
UHPC	F05R00	0.5	0
	F10R00	1.0	0
R-UHPC	F05R17	0.5	1.7
	F05R30	0.5	3.0
	F05R47	0.5	4.7
	F05R68	0.5	6.8
	F10R17	1.0	1.7
	F10R30	1.0	3.0
	F10R47	1.0	4.7
	F10R68	1.0	6.8

**Table 4:** Tensile mechanical properties of R-UHPC with 0.5% fiber volume fraction.

Specimens	F05R17	F05R30	F05R47	F05R68
Cracking load *F*_cr_ (kN)	19.4 ± 0.07	17.9 ± 3.0	14.3 ± 0.9	13.5 ± 2.1
Cracking strain *ε*_cr_ (%)	0.007 ± 0.001	0.007 ± 0.000	0.005 ± 0.000	0.005 ± 0.001
Yield load *F*_y_ (kN)	53.0 ± 1.3	87.7 ± 1.2	125.0 ± 5.3	157.8 ± 1.8
Yield strain *ε*_y_ (%)	0.418 ± 0.001	0.476 ± 0.019	0.482 ± 0.008	0.477 ± 0.020
Peak load *F*_p_ (kN)	62.4 ± 1.2	100.0 ± 3.0	152.0 ± 0.0	201.1 ± 0.4
Peak strain *ε*_p_ (%)	1.804 ± 0.385	4.436 ± 0.084	12.845 ± 1.027	25.941 ± 0.395
Ultimate load *F*_u_ (kN)	53.0 ± 1.0	85.0 ± 2.5	129.2 ± 0.0	171.0 ± 0.4

Note: The data are expressed in the format: mean ± standard deviation.

**Table 5:** Tensile mechanical properties of R-UHPC with 1.0% fiber volume fraction.

Specimens	F10R17	F10R30	F10R47	F10R68
Cracking load *F*_cr_ (kN)	20.1 ± 3.6	21.0 ± 2.1	16.6 ± 1.8	17.1 ± 1.0
Cracking strain *ε*_cr_ (%)	0.006 ± 0.002	0.006 ± 0.001	0.005 ± 0.000	0.005 ± 0.000
Yield load *F*_y_ (kN)	61.8 ± 1.8	96.5 ± 0.7	134.2 ± 0.7	170.1 ± 1.0
Yield strain *ε*_y_ (%)	0.366 ± 0.013	0.397 ± 0.010	0.412 ± 0.016	0.427 ± 0.022
Peak load *F*_p_ (kN)	72.9 ± 2.2	108.4 ± 1.3	152.5 ± 1.0	201.2 ± 0.3
Peak strain *ε*_p_ (%)	1.091 ± 0.150	1.144 ± 0.215	10.700 ± 1.192	24.232 ± 0.353
Ultimate load *F*_u_ (kN)	62.0 ± 1.9	92.2 ± 1.1	129.6 ± 0.8	171.0 ± 0.3

Note: The data are expressed in the format: mean ± standard deviation.

## Data Availability

Data are contained within the article.
